# Supplementary material for: Splice-Junction-Based Mapping of Alternative Isoforms in the Human Proteome
Source: Cell Rep. Author manuscript; Available in PMC 2020 Jan 15. (PMC6961840; doi:10.1016/j.celrep.2019.11.026)
Supplement: 3 [file NIHMS1546469-supplement-3.zip › DF2/PXD009737/TestisTrypsin-423-Q15032-NEHCIENNIDLQEK.pdf]

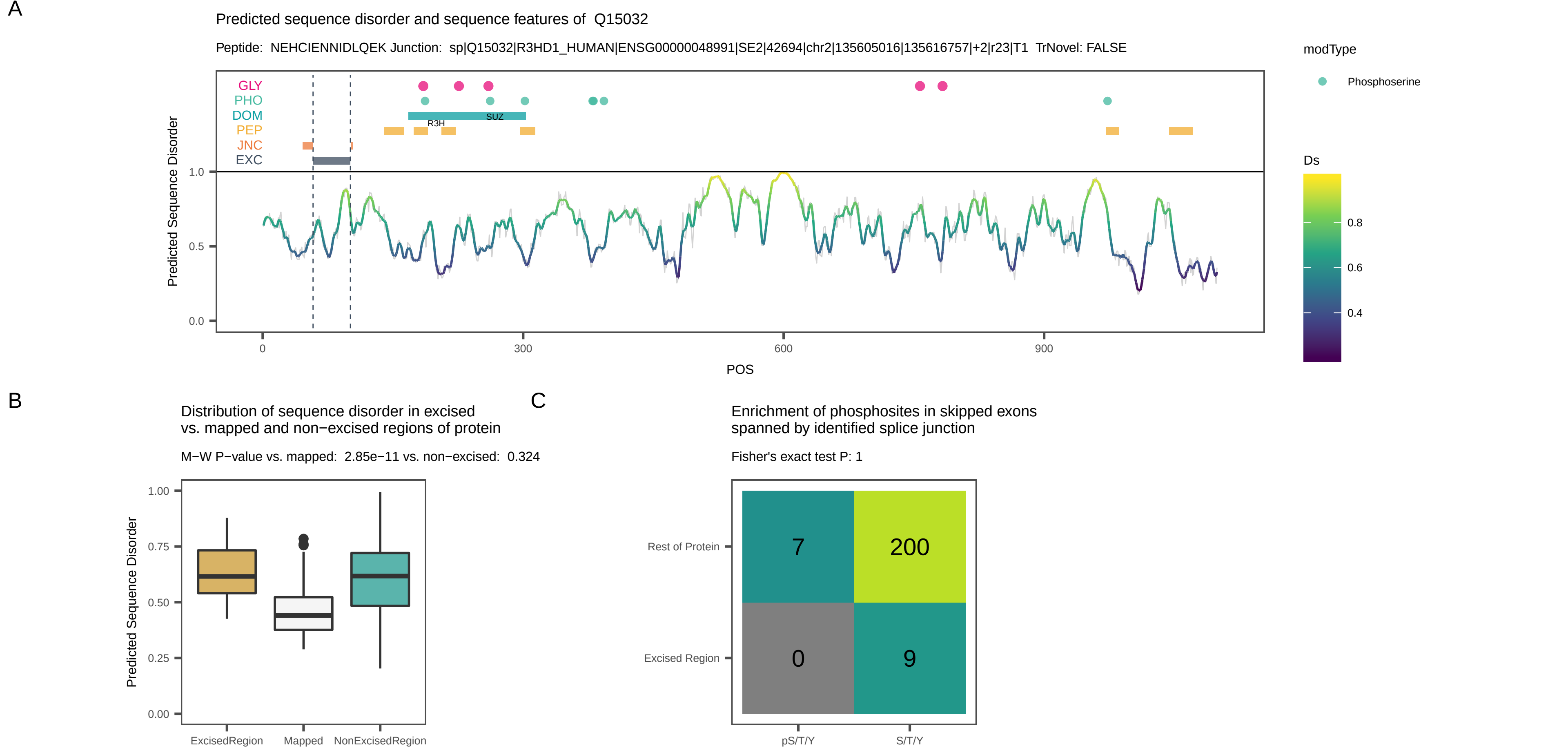
A
Predicted sequence disorder and sequence features of Q15032
Peptide: NEHCIENNIDLQEK Junction: sp|Q15032|R3HD1_HUMAN|ENSG00000048991|SE2|42694|chr2|135605016|135616757|+2|r23|T1 TrNovel: FALSE
GLY
PHO
DOM
PEP
JNC
EXC
R3H
SUZ
Predicted Sequence Disorder
1.0
0.5
0.0
0
300
600
900
POS
modType
Phosphoserine
Ds
0.8
0.6
0.4
B
Distribution of sequence disorder in excised vs. mapped and non−excised regions of protein
M−W P−value vs. mapped: 2.85e−11 vs. non−excised: 0.324
Predicted Sequence Disorder
1.00
0.75
0.50
0.25
0.00
ExcisedRegion
Mapped
NonExcisedRegion
C
Enrichment of phosphosites in skipped exons spanned by identified splice junction
Fisher's exact test P: 1
Rest of Protein
Excised Region
7
200
0
9
pS/T/Y
S/T/Y
